# NK Cells in Myeloproliferative Neoplasms (MPN)

**DOI:** 10.3390/cancers13174400

**Published:** 2021-08-31

**Authors:** Erin Naismith, Janine Steichen, Sieghart Sopper, Dominik Wolf

**Affiliations:** 1 Internal Medicine V, Department of Haematology and Oncology, Medical University Innsbruck, Anichstr. 35, 6020 Innsbruck, Austria; erin.naismith@i-med.ac.at (E.N.); janine.steichen@i-med.ac.at (J.S.); 2 Tyrolean Cancer Research Institute, Innrain 66, 6020 Innsbruck, Austria

**Keywords:** innate immunity, CML, polycythemia vera, essential thrombocythemia, primary myelofibrosis

## Abstract

**Simple Summary:**

NK cells are important innate immune effectors that contribute substantially to tumor control, however the role of NK cells in haematological cancers is not as well understood. The aim of this review is to highlight the importance of the role of NK cells in the management of Ph+ Myeloproliferative Neoplasms, and emphasize the need and possible benefits of a more in-depth investigation into their role in classical MPNs and show potential strategies to harness the anti-tumoral capacities of NK cells.

**Abstract:**

Myeloproliferative neoplasms (MPNs) comprise a heterogenous group of hematologic neoplasms which are divided into Philadelphia positive (Ph+), and Philadelphia negative (Ph−) or classical MPNs. A variety of immunological factors including inflammatory, as well as immunomodulatory processes, closely interact with the disease phenotypes in MPNs. NK cells are important innate immune effectors and substantially contribute to tumor control. Changes to the absolute and proportionate numbers of NK cell, as well as phenotypical and functional alterations are seen in MPNs. In addition to the disease itself, a variety of therapeutic options in MPNs may modify NK cell characteristics. Reports of suppressive effects of MPN treatment strategies on NK cell activity have led to intensive investigations into the respective compounds, to elucidate the possible negative effects of MPN therapy on control of the leukemic clones. We hereby review the available literature on NK cells in Ph+ and Ph− MPNs and summarize today’s knowledge on disease-related alterations in this cell compartment with particular focus on known therapy-associated changes. Furthermore, we critically evaluate conflicting data with possible implications for future projects. We also aim to highlight the relevance of full NK cell functionality for disease control in MPNs and the importance of considering specific changes related to therapy in order to avoid suppressive effects on immune surveillance.

## 1. Introduction

### 1.1. Introduction to MPNs

The group of MPNs belong to the family of chronic myeloid neoplasms and comprise a variety of clonal hematological diseases, characterized by the increased proliferation of cells originating from the myeloid lineage [1,2,3,4,5]. Typically, the group of MPNs is subdivided into two major groups: the Philadelphia chromosome carrying CML and the classical or Ph− MPNs [6]. Summarized under the term classical MPNs are polycythemia vera (PV), essential thrombocythemia (ET), primary myelofibrosis (PMF), chronic neutrophilic leukemia (CNL) and chronic eosinophilic leukemia—not otherwise specified (CEL-NOS) [5]. Annual incidence rates of PV, ET, PMF and CML are around 1–2/100.000 whereas CNL and CEL-NOS are extremely rare conditions [6,7,8,9,10].

All MPN entities originate from a single mutated hematopoietic stem cell (HSC) with subsequent proliferation through clonal expansion [4]. Depending on disease entity and main disease-driving mutation, clonal expansion can affect single or multiple cell lineages [4]. The respective somatic driver mutations are then found among all myeloid lineages; however, they may also be detectable at lower frequencies within the lymphoid lineage, particularly in B and NK cells [4].

#### 1.1.1. Disease Characteristics

MPNs include a variety of subtypes with numerous gene alterations involved, resulting in a heterogenous landscape of phenotypes. Accurate diagnosis of the respective disease entities therefore requires careful consideration of clinical and morphological features as well as the genetic aberrations involved [2,4,5,11,12].

#### 1.1.2. Ph+ MPNs

The most prominent of the somatic alterations known in MPNs is the *BCR-ABL1* gene rearrangement, constituting an exclusive feature of CML [13,14]. Due to the disease-defining reciprocal translocation t(9;22) (q34;q11), termed the Philadelphia chromosome, CML is considered a unique entity and model disease among the group of MPNs [13,15]. From a clinical perspective, CML often presents in chronic phase with symptoms ranging from mild and unspecific to asymptomatic [13,15]. Without accurate treatment, the disease naturally progresses over the accelerated phase into blast crisis in the course of 3–5 years [13,15]. This serious condition ultimately leads to patient death by means of thrombosis, anemia or infection [13]. However, with modern therapeutic standards and the introduction of tyrosine kinase inhibitors (TKIs) the life expectancy of CML patients has been significantly ameliorated and almost meets the general population’s [16].

#### 1.1.3. Classical MPNs

Among the Ph− MPNs, the main clinical manifestations differ substantially and largely depend on the respective entity’s most prominent hematologic features. All MPNs can furthermore vary in disease severity and thus show a wide range of symptom intensity. PV patients may present with marked erythrocytosis and associated concomitant symptoms of hyperviscosity, microvascular symptoms and bleeding complications, whereas classical ET is characterized by an elevation in platelet count and history of thrombosis [1,17,18,19]. A typical sign of PMF is bone marrow fibrosis accompanied by marked anemia, cachexia and thrombohemorrhagic events [20,21]. A hallmark finding of CNL is an extensive bone marrow neutrophilic granulopoiesis, while CEL-NOS presents with typical hypereosinophilia with eosinophil clonality [9,10]. Frequently occurring mutated genes in PV, ET and PMF include *JAK2*, *CALR* and *MPL*. These mutations all ultimately result in increased downstream activation of the STAT and other disease-promoting pathways [4,18,19,20,22]. The mentioned classical genetic alterations are however absent in CNL and CEL-NOS [9,10].

## 2. Immunological Changes in MPNs

Immune evasion by tumor-associated immune dysregulation is a phenomenon that was detected in a variety of tumor entities [23]. Furthermore, the concept of oncoinflammation has given rise to the idea that a hyperinflammatory tumor microenvironment may also contribute to tumor development and progress [23,24].

Classical myeloproliferative diseases are also driven by inflammation and the excessive activation of inflammatory pathways has several immunomodulatory effects [25,26,27]. Increased pro-inflammatory cytokines and the accumulation of reactive oxygen species as well as the transcriptional deregulation of anti-oxidative stress genes were found in patients with JAK2^V617F^ mutant MPN [28,29]. This up-regulation has been reported to stimulate the JAK/STAT signaling axis, resulting in an increased proliferation of various cell types, including an increased viability and survival of malignant cancer cell clones [30]. Studies in mice have shown that administering the anti-oxidant N-acetylcysteine (NAC), could reduce splenomegaly and the number of JAK2-mutant hematopoietic stem and progenitor cells in the spleen and BM, and normalize blood parameters [28].

With the discovery that CML patients can potentially stay in treatment free remission (TFR) even after treatment cessation, it became clear that the immune system must play a pivotal role in disease control of Ph+ MPNs [31,32]. Numerous findings have been reported, showing that immunological variables serve as predictors of molecular response and depth of remission, and thus support the hypothesis of a close interaction between the immune system and survival of the leukemic clone [33]. Current data suggest that the restoration of immune-effectors, particularly involving T and NK cells, are key-points to achievement of molecular response and durable TFR [33,34].

## 3. Natural Killer Cells

Natural killer (NK) cells are innate lymphocytes with effector functions, they play an important role in host defense and immune surveillance and are known for their ability to rapidly kill tumor- and virus-infected cells. NK cells also produce cytokines such as IFN-γ and TNF-α, which play an important role in the differentiation and maturation of both innate and adaptive immune cells [35]. Murine models have shown that NK cell development is complex and tightly regulated, and while human NK cells are generated and maintained by HSCs, the precise process is not yet fully elucidated [36,37]. The main site of NK cell generation is considered to be the immunological niche in the BM; however, whether the NK cell ontogenesis occurs exclusively in the BM niches remains debated, as evidence suggests that immature NK cells can migrate to secondary lymphoid tissue to mature [38]. IL-15 is produced by stromal cells in the BM and is important for the differentiation and survival of NK cells. As common lymphoid progenitors develop into NK progenitors, they down regulate CD34 and acquire the expression of the IL-15 receptor (IL-15R). Various other chemokines and receptors are expressed on the different subsets of NK cells, influencing their tissue localization and regulating their release from the BM [39]. Different anatomical sites and tissues possess different homeostatic mechanisms, which result in tissue specific NK cell development, and maintain the balance between immune tolerance and immune surveillance [36].

The diversity of the NK cell repertoire is determined by the large array of activating and inhibitory receptors expressed on the cell surface. Inhibitory receptor expression is intrinsic, encoded by the host germline, while activating receptors can be influenced by extrinsic factors such as epigenetics or chronic infection [40,41]. These activating and inhibitory markers form a delicate balance on the cell’s surface, determining whether the NK cell will be activated upon presentation of the target cell [42].

Unlike T cells, NK cells do not require any previous priming and they exert anti-metastatic functions via (1) the release of lytic granules containing perforin and granzymes, (2) IFN-γ secretion, and (3) the exposure of death receptor ligands, e.g., TRAIL and FASL, which when bound, can induce apoptosis [43].

Under homeostatic conditions, they remain in a resting state due to inhibitory markers such as inhibitory Killer Immunoglobulin-like receptors (iNKRs), C-type lectin receptor NKG2A, Ig-like transcripts (ILTs), and leukocyte Ig-like receptors (LIRs). These inhibitory receptors recognize a wide range of Human Leukocyte Antigens (HLAs) which allow autologous cells to be identified as “self” [36]. The dearth or downregulation of HLA-I alleles by infected or tumor cells boosts NK cell-mediated killing, engaging various NK cell activation receptors (aNKRs), a concept referred to as the “missing-self” hypothesis [44]. aNKRs include Natural Cytotoxicity Receptors (NCRs) such as NKp46, C-type lectin receptors such as NKG2D, DNAM-1 and Killer Ig-like Receptors (KIRs) [36,37]. aNKR ligands can also be upregulated by cellular stress or as a response to DNA damage, both of which are common in many different types of cancer [45]. The secretion of soluble NKG2D ligands (MICA and MICB) by cancer cells, has been postulated as a possible mechanism of NK cell killing evasion. MICA and MICB are able to downregulate NKG2D on the NK cell without triggering activation [46]. Indeed, sMICA levels were shown to be increased in CML patients and to return to normal levels with imatinib therapy [47].

The phenotype and density of the cell surface antigen expression is theorized to explain a functional distinction between the subsets [48]. The two main subsets of NK cells can be defined by their surface expression of CD56 and CD16. CD56, also known as neural cell adhesion molecule (NCAM), plays an important role in adhesion in bone marrow niches [49] and CD16 is the type III Fcγ receptor which binds to the Fc region of antibodies and triggers the release of cytotoxic granules [50]. CD56^dim^CD16^+^ cells are cytotoxic NK cells and make up the majority of circulating NK cells (~90%). CD56^bright^ CD16^−^ NK cells are defined as a regulatory subset, producing high levels of pro-inflammatory cytokines [36,51]. The vast array of NK cell surface receptors is complex and distinct within the different sub-populations, highlighting the functional heterogeneity of NK cells [48]. Environmental factors, including pathogen exposure or the microenvironment in which the cell develops and resides, may also influence receptor diversity [36].

NK cells are pivotal in the control of metastatic dissemination of solid tumors and an inverse correlation has been observed between circulating NK cells and metastases at clinical presentation in a range of different carcinomas, while improved NK cell cytotoxicity has previously been linked with good prognosis [52]. NK cells have been described as ‘critical’ to immunosurveillance and anti-tumor activity in B cell lymphomas [53], chronic myeloid leukemia (CML), acute myeloid leukemia (AML), and myelodysplastic syndromes (MDS) [54], highlighting the importance of further elucidating their role in hematological cancers.

## 4. NK Cells in MPN

### 4.1. CML

#### 4.1.1. NK Cells in CML

**Figure 1:**
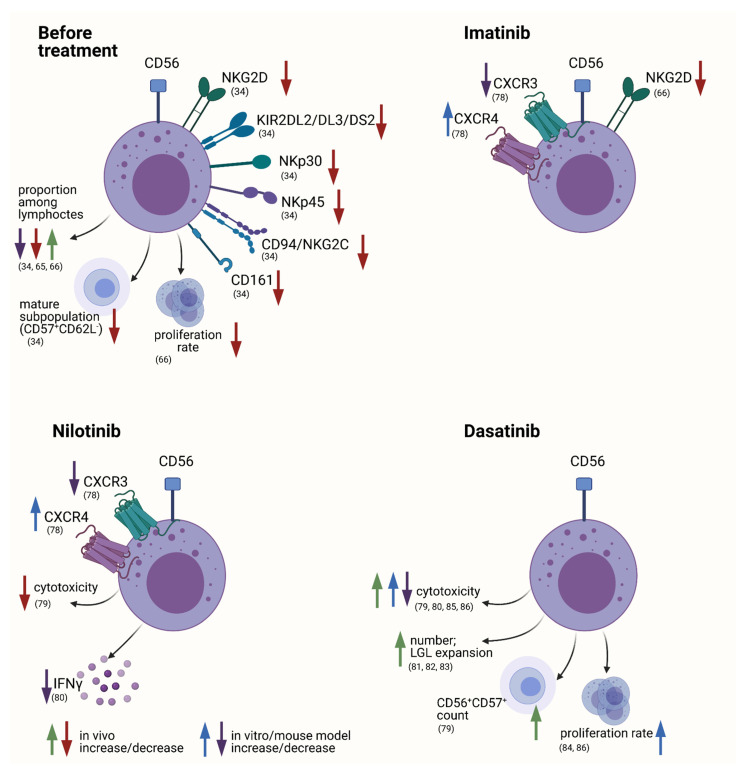
Phenotypic and functional alterations in the NK cell compartment of CML patients before therapy initiation, as well as TKI-specific changes. An in vivo increase or decrease is displayed with green or red arrows respectively, while blue and purple arrows indicate an observed increase or decrease in vitro or using a mouse model. References are given in brackets. Created with BioRender.com (accessed on 15 June 2021).

A considerable amount of the immunological changes and influencing factors in CML concern phenotypic and functional alterations of the NK cell compartment which makes them an interesting topic for closer investigation [55] (Figure 1).

T/NK progenitors derived from untreated chronic phase CML patients, were shown to express the Philadelphia chromosome [56]. Interestingly, the vast proportion of mature NK cells were reportedly negative for *BCR-ABL1* [56,57,58]. This discrepancy in Philadelphia positivity is explained by a lack of malignant precursors to differentiate into mature NK cells [59]. In advanced phases of CML, however, both CD56^dim^ and CD56^bright^ populations can become Ph+ and can thus contribute to the malignant clone [58,59]. Although, in chronic phases of CML, most peripheral NK cells are not directly part of the disease itself, they have, as important key players of immunity, been subjected to comprehensive investigation in order to obtain a better understanding of immunologic surveillance in CML. 

The first findings demonstrating that NK cells are capable of killing leukemic blasts derived from CML patients, date back to 1983 [60]. Reports by Lotzová et al. confirmed the capability of NK cells to inhibit the growth of leukemic cells in CML [61]. Cervantes et al. furthermore demonstrated that NK cells derived from healthy donors, as well as from CML patients, exhibited cytotoxic activity towards the cell line K562 in vitro, and that this effect was mediated by direct cell-to-cell contact [62]. Cebo et al. supported this finding by showing that lysis of BCR-ABL1 expressing cell lines by NK cells was mediated by an interaction of NK-receptor NKG2D and its corresponding ligands [63]. Primitive, quiescent CD34^+^ progenitor cells derived from CML patients were the least susceptible to NK cell cytotoxicity in vitro [61,64].

NK cells therefore seem capable of killing the leukemic clone in CML and may play a crucial role in disease control. Various findings have demonstrated a decrease in quantity, as well as functional deficits, of NK cells in CML [54]. However, these assumptions were followed by contradictory findings, indicating a more complex situation [54,65].

##### General Alterations in the NK Cell Compartment

Several studies reported reduced relative amounts of NK cells within the lymphocytic compartment in newly diagnosed CML patients compared to healthy controls [34,66]. The decrease of NK cell proportions remained detectable even under imatinib treatment with stable remission in a study by Chen et al. [66]. Opposing data, however, demonstrated a significant increase of NK cell numbers with consequent TKI therapy, in particular with dasatinib treatment [34,67].

Chen et al. investigated the finding of NK cell reduction in newly diagnosed CML using a transgenic mouse model with reversible *BCR-ABL1* expression [66]. They found that the relative proportions of murine NK1.1^+^ were significantly reduced with *BCR-ABL1* induction and remained low even after *BCR-ABL1* reversion [66]. Upon functional exploration, the group demonstrated that the murine NK cells isolated from *BCR-ABL1*^+^ mice did not show any difference in proliferative activity, whereas the degranulation capacity was significantly decreased when the mutation was expressed [66]. However, in contrast to findings in human studies, it should be highlighted that in this experimental setting, NK cells expressed the *BCR-ABL1* transcript [66].

We have previously demonstrated that assumptions regarding NK cell numbers in CML must be interpreted cautiously [65]. When quantifying the lymphocytic compartment in this entity it is of utmost importance to take into account the high number of basophil and hematopoietic progenitors that rapidly normalizes with TKI administration. As lymphocytes, basophils and progenitors all have similar SSC and FSC characteristics in flow cytometry, relative numbers of lymphocytes such as NK cells will be underestimated at time of diagnosis. It is therefore crucial to first exclude the abundant cell types of basophils and progenitors. We have thus defined lymphocytes in whole blood via flow cytometry using the different levels of CD45 for exclusion of the aforementioned cell populations among the FSC and SSC defined lymphocyte gate. While other groups performed stainings on PBMCs, we used whole blood to obtain more accurate absolute numbers. With these improvements, we found, in contrast to previous reports, a significantly higher number of NK cells (including CD56^dim^ and CD56^bright^) at time of diagnosis compared to months 6 and 12 after treatment initiation with nilotinib [65]. This was true for both percentages of lymphocytes, as well as for absolute numbers [65].

In addition to the changes in quantity, phenotypic alterations and changes in the composition of the NK cell compartment have been reported. Hughes et al. demonstrated a significant decrease, in proportions of lymphocytes, of the cytokine-producing CD56^bright^ cells, as well as the CD56^dim^ group at diagnosis compared with patients in molecular remission [34]. They also found a significant reduction of a mature NK subpopulation (defined as CD57^+^CD62L^−^) at CML diagnosis [34]. Chen et al. showed that, even with the total NK cell proportions among lymphocytes being reduced in CML at diagnosis, concerning the CD56^dim^ and CD56^bright^ subpopulations, there was no difference in composition between CML patients and healthy individuals [66].

Further reports have described a loss of activating NK cell receptors at time of diagnosis, such as NKG2D, CD94/NKG2C, CD161, NKp30, NKp46 or KIR2DL2/DL3/DS2 [32,34,68]. Additionally, expression of the activating KIR receptor, KIR2DS1, was shown to be increased in CML patients undergoing TKI therapy compared with healthy individuals [69]. The KIR-ligand combination KIR2DS2/KIR2DL2 absent/HLA-C1 present was also significantly reduced in CML patients [69].

The aforementioned decrease of NK cell activating receptors may consequently cause an imbalance between activating and inactivating receptors and confer suppression of NK cell activity. Downregulation of receptor NKG2D or loss of the correlating ligands, is thought to contribute to immune escape of the leukemic clone by decreasing the NK cell mediated recognition of *BCR-ABL1* positive cells [32,63,70]. In another report, however, NKG2D positive NK cell proportions were similar between healthy individuals and CML patients before treatment initiation [66].

In addition to the phenotypic changes mentioned—or resulting from them—NK cells in Ph+ MPN were described to display functional deficits, which may inhibit sufficient control of the leukemic clone. These alterations were discovered not only at baseline but intriguingly also during therapy.

Chen et al. demonstrated NK cell dysfunction in vitro [66]. Incubation of NK cells derived from newly diagnosed CML patients with the K562 cell line resulted in significantly lower degranulation, which persisted in patients undergoing imatinib therapy [66]. Furthermore, they showed that NK cells derived from CML patients, both before therapy initiation and under imatinib-mediated remission, had a significant reduction in proliferation rate using a 10-day expansion protocol with K562 and IL-2 incubation compared with healthy individuals [66]. However, this stands in contrast to findings of normal NK cell proliferative activity demonstrated in the group’s *BCR-ABL* expressing mouse model, as mentioned above [66]. It remains to be elucidated whether these discrepancies are due to the fact that NK cells derived from the mouse model expressed the *BCR-ABL* transcript, or can be explained by other experimental variables [66] (Figure 1).

##### Specific Changes during Course of Treatment with Possible Functional Consequences

Treatment of CML is primarily dictated by the characteristic *BCR-ABL1* mutation and with the introduction of imatinib in 2000, mainly consists of single-agent TKI administration [16]. These TKIs target the *BCR-ABL1* transcript itself and inhibit its tyrosine kinase function [71]. In the past years, 2nd generation TKIs, namely dasatinib, nilotinib and bosutinib, have proven higher efficacy with deeper molecular remissions in first- and further lines of treatment, compared to 1st generation TKI imatinib [72]. The 3rd generation TKI ponatinib may be indicated in case of resistance to prior lines and/or T314I mutation [72]. Later phases of CML, namely the advanced or blast phase, may even necessitate further treatment escalation and an allogeneic stem cell transplantation (allo-SCT) could be required as a final therapeutic option [72]. When deep remissions are achieved with TKI treatment, CML patients can ultimately become eligible for treatment cessation and experience treatment-free remission (TFR), meaning stable molecular remission without further specific therapy [72].

Single-agent treatment with IFN-α has disappeared from the therapeutic landscape of CML due to modern TKI regimes. Nevertheless, it is still under investigation as a possible add-on to current therapies and thus exploration of alterations in the NK cell compartment under IFN-α treatment are still of interest. In a study by Kreutzman et al. comparing patients under IFN-α monotherapy with patients after successful IFN-α discontinuation, NK cell proportions were considerably higher in the patient cohort after stopping IFN-α treatment compared with patients still under treatment and healthy individuals [73]. As the study had no long-term data available, it remained elusive to what extent the decrease of NK cell proportions was a consequence of IFN-α application or whether low NK cell counts were a negative prognostic factor for successful discontinuation. Ilander et al. similarly demonstrated that relative NK cell numbers among lymphocytes were significantly higher in patients after IFN-α monotherapy discontinuation compared to patients under treatment and healthy controls, however, this finding was not statistically significant concerning absolute NK cell numbers [74]. Mature NK cells (CD56^dim^CD62L^low^CD27^low^CD57^+^) were more abundant in patients after successful discontinuation of IFN-α therapy [74]. Furthermore, activated NK cells (CD56^+^HLA-DR^+^) were shown to be increased in patients with complete hematologic remission still undergoing IFN-α monotherapy [75]. Concurrently, in these patients NK cell cytotoxicity against cell line K562 was increased together with an increase in FasL expression on NK cells, which was suggested to help control the Fas expressing CD34^+^ stem cells [75]. The combination of TKIs with IFN-α led to an increase in the amount of CD56^bright^ NK cells compared to TKI therapy alone [76].

Similarly, modern TKI therapy in CML patients has shown to exert various immunomodulatory effects, not overlooking the NK-cell compartment (Figure 1) [32]. The specific differences among the types of TKIs may be at least partly explained by the pattern of kinases and additional off-target effects that are inhibited in addition to BCR-ABL1 with each respective drug [77,78]. First reports of impairment of NK cell function in vitro, especially by dasatinib and nilotinib, led to extensive investigation of possible NK cell-modulating mechanisms [79].

In vitro experiments conducted by the group of Salih et al. showed that the three TKIs imatinib, nilotinib and dasatinib led to a significant reduction of MICA and MICB on the K562 cell line [80]. These cell surface proteins are ligands of NK cell receptor NKG2D and are considered important for the detection of CML cells [80]. The functional impact of this downregulation was confirmed in co-culture experiments of NK cells with 24h treated K652 cells [80]. The group was able to demonstrate that the loss of MICA and MICB led to reduced IFN-γ production, as well as to a decrease in NK cell cytotoxicity [80]. Confirmatory findings from Boissel et al. and Cebo et al. also demonstrate the downregulation of MICA/B expression on *BCR-ABL1^+^* cells treated with imatinib in vitro [47,63]. They furthermore confirmed the reduced NKG2D dependent lytic capacity towards BCR-ABL1 expressing cell lines after imatinib application [47,63].

Salih et al. however, found no direct effect of in vitro imatinib administration on NK cell reactivity, as NK cell cytotoxic capacity and cytokine production were not affected by immediate addition of the substance without pre-incubation of K562 with imatinib [80]. There was also no effect of in vitro imatinib application concerning cytotoxicity or cytokine production when primary material derived from CML patients before treatment was used as the NK cell target [80]. Furthermore, in vivo treatment with imatinib did not lead to changes in the lytic capacity of CML-derived NK cells compared to healthy controls [79]. 

Chen et al. have also comprehensively characterized the NK cell compartment in CML patients under imatinib therapy and showed that the expression of activating NK cell receptor NKG2D was significantly decreased with imatinib therapy [66]. As NKG2D is known to be an activating NK cell receptor this finding would indicate that imatinib application could potentially decrease leukemic cell control [66]. Chen et al. however, were able to demonstrate, similarly to Salih et al., that addition of imatinib to degranulation assays in vitro had no functional consequences on either expanded NK cells derived from healthy individuals or from newly diagnosed CML patients [66].

Bellora et al. have further investigated phenotypic alterations of NK cells in vitro with imatinib and nilotinib administration [78]. With application of both substances respectively, they found a decrease in CXCR3 with CXCR4 upregulation [78]. It was hypothesized that this combination may inhibit successful recruitment of NK cells, as CXCR3 is important in chemotaxis whereas CXCR4 is involved in NK cell homing and the preservation of the cells in the bone marrow niche [78].

Upon investigation of the effect of nilotinib on functionality of polyclonal NK cells towards cell line K562 or primary PBMCs derived from CML patients, Salih et al. found no substantial decrease in cytotoxicity, but a decline in IFN-γ production [80]. Furthermore, upon incubation of NK cells derived from healthy donors with nilotinib, the group demonstrated an increase of dead cells in the CD56^bright^ NK cell subset, with no effect on the cytotoxic CD56^dim^ compartment [80]. As the CD56^bright^ compartment is the major cytokine-producing NK cell population, this could partly explain the decrease in IFN-γ production [80]. In contrast to these in vitro results, Hayashi et al. found a decrease in cytotoxic activity of NK cells derived from patients under nilotinib treatment [79]. These discrepancies may be the result of different investigation methods, as Salih et al. investigated the direct effect of nilotinib administration as an in vitro effect on NK cells, whereas Hayashi et al. demonstrated the decrease in lytic capacity in nilotinib-treated patient samples. Furthermore, it may be possible that in Hayashi’s cohort, the lytic activity of NK cells was already diminished a priori, and it was not restored with nilotinib treatment.

During dasatinib treatment, a characteristic expansion of so-called large granular lymphocytes (LGL), comprising of CD8^+^ T-cells and NK cells, was previously described in vivo [32,81,82]. This finding is specific for dasatinib among the group of TKIs [81,82]. As cytotoxic T cells, as well as NK cells, might contribute to antitumor immunity in CML, the observed expansion of LGL may constitute a beneficial characteristic of dasatinib treatment [82].

Moreover, the D-first study, an open-label study on dasatinib, demonstrated that general lymphocytosis, defined by differential blood count, was a frequent event to occur in approximately 27% of patients by the 18 month mark [83]. Among these, NK cell counts were also found to increase with dasatinib treatment [83]. Hara et al. reported a dose-dependent augmentation of NK-cell numbers derived from healthy volunteers with in vitro dasatinib application [84].

Concerning the functional exploration of dasatinib on degranulation and cytotoxicity of NK cells, contradictory results have demonstrated the importance of a clear definition of the experimental setups comparing with- versus without- “wash-out” settings. This is especially necessary in vitro when the drug is added directly to the incubational assay. In a study conducted by Salih et al., dasatinib significantly reduced cytotoxicity and IFN-γ production in vitro [80]. Dasatinib administration also led to comparable short-term and reversible dose-dependent reduction of cytotoxicity, degranulation and cytokine secretion of NK cell lines in a study performed by Hassold et al. when no dasatinib wash-out was performed [85]. Simultaneously however, in the same study stimulatory effects were seen applying experimental settings with dasatinib pre-treatment and subsequent wash-out before functional investigation [85]. This finding suggests that the wash-out of the drug is crucial when clearly defining the long-term effects of dasatinib in vitro and more adequately mimicking in vivo conditions.

A confirmatory finding was provided in a study performed by Uchiyama et al. [86] Dasatinib administration to peripheral blood mononuclear cells (PBMCs) in vitro derived from healthy donors led to a dose-dependent increase in proliferation of NK cells (both in percentages and absolute counts) as well as to elevation of cytolytic activity using K562 target cells when dasatinib wash-out was performed [86].

Further investigations validating the stimulatory effect of dasatinib were provided by Hayashi et al. [79]. The group demonstrated that NK cells derived from patients under dasatinib treatment had an increase in cytotoxic reactivity [79]. Interestingly, they also found an increase in NK cell number with in vivo dasatinib treatment, especially the CD56^+^CD57^+^ compartment, which was simultaneously associated with higher NK cell-specific cytotoxic activity [79] (Figure 1).

With the current ongoing pandemic, it is interesting to note that the Italian Campus CML program has reported low incidences of COVID-19 infections in CML patients undergoing TKI treatment [87]. As NK cells are considered to be important players in infection control of COVID-19, it may be assumed that under successful TKI therapy NK cells are functional enough to exert antiviral characteristics [88].

##### The Role of NK Cells to Disease Progression and Response to Therapy

**Figure 2:**
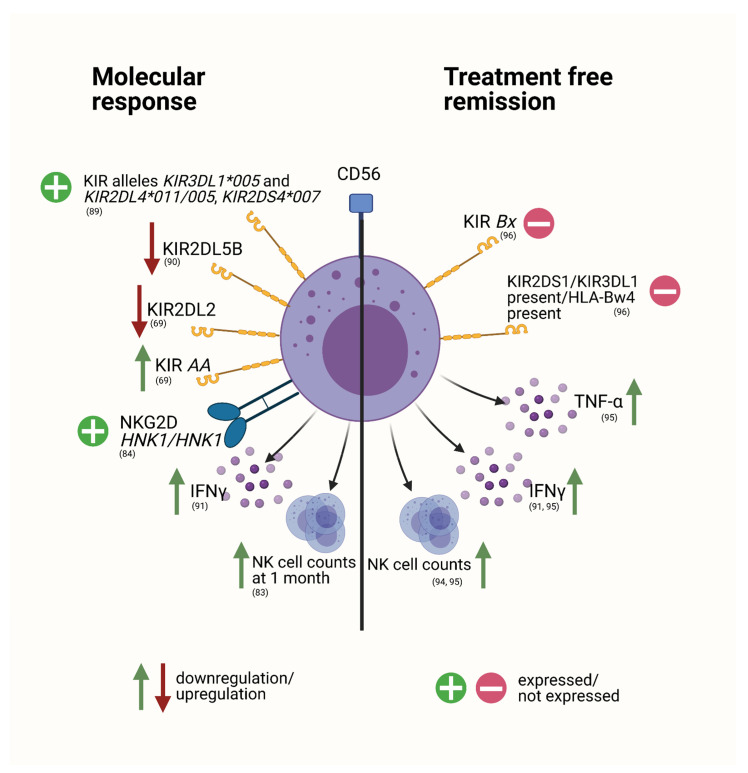
NK-cell associated changes serving as prognostic factors for molecular response or treatment free remission in CML. Green arrows indicate a favorable effect of an increase in the respective marker, while red arrows demonstrate that a decrease is beneficial to achieving molecular remission or treatment free remission. A circled green cross or red minus indicate a positive prognostic value of either the expression or absence respectively, of a certain marker. References are shown in brackets. Created with BioRender.com (accessed on 15 June 2021). * refers to the genetic mutation.

In addition to the changes conferred by therapeutic interventions in CML, several studies assessing NK cell related parameters have also found prognostically relevant associations with response to TKI therapy (Figure 2).

A comprehensive investigation by Ureshino et al. on the predictive role of the inhibitory receptor KIR3DL1 and the associated HLA-B allelic polymorphisms has revealed that weakly interacting combinations of these two counterparts were associated with a superior response to TKI treatment [89]. Alleles KIR3DL1*005 and KIR2DL4*011/005 as well as KIR2DS4*007 also conferred favorable prognostic value [89]. A sub-study of the TIDEL-II study, a trial examining a risk-adapted scheme of imatinib and nilotinib combination, found the expression of KIR2DL5B to be an independent negative prognostic factor for achievement of MMR (major molecular remission) and was furthermore significantly linked to inferior achievement of MR^4.5^, event-free survival (EFS) and transformation-free survival (TFS) [90]. As KIR2DL5B is an inhibitory KIR receptor, it was consequently hypothesized that its expression could possibly suppress sufficient NK-cell mediated killing of leukemic cells [90]. Loss of the inhibitory receptor KIR2DL2 was associated with successful CMR (complete molecular remission) in a heterogeneously treated patient population under 1st and 2nd generation TKI therapy as reported by Nasa et al. [69]. In the same cohort, KIR genotype AA was linked to earlier CMR, with a higher probability of CMR achievement in comparison to genotype Bx [69]. KIR genotype AA thus appeared to be a strong predictive factor for successful CMR in this setting [69].

The amount of effector NK cells (defined as IFN-γ^+^ NK cells) has also been previously linked to successful and stable CMR under imatinib treatment [91]. The increase in this cell compartment may therefore constitute a marker for functioning immune surveillance [91]. In a patient population under dasatinib treatment, Iriyama et al. demonstrated that NK cell counts at 1 month after treatment initiation revealed to be a crucial predictive factor for DMR [83]. This suggests that a rapid increase in NK cell count, potentially caused by dasatinib itself, may reflect a successful immune response towards leukemic cells [83]. Additionally, a study by Hara et al. revealed that expression of NKG2D *HNK1/HNK1* haplotype was linked to a higher likelihood of achieving MR^4.5^ in dasatinib treated patients [84] (Figure 2).

It is therefore likely, that NK cells have a potential role in control of the leukemic clone, even in patients with ongoing TKI treatment. Furthermore, various reports indicate that NK cells also seemingly play a pivotal role in the achievement and maintenance of successful TFR [92].

##### NK Cells in the Setting of Treatment Discontinuation

In the past years the concept of treatment discontinuation with consequent TFR has become a desirable and achievable goal in CML therapy [92]. However, TFR is not always successful. One potential reason for discrepancies in TFR achievement are differences in immune response (Figure 2) [92].

The IMMUNOSTIM trial group demonstrated that higher numbers of peripheral CD56^dim^ NK cells at the time of treatment cessation were significantly and independently correlated to treatment-free remission after imatinib discontinuation [93]. In agreement with these results are the EUROSKI and DADI trials on imatinib and dasatinib treatment cessation respectively, also demonstrating the protective effect of NK cell increase in the setting of TFR [94,95].

The IMMUNOSTIM group furthermore found no differences in expression of an extensive panel of NK cell receptors comparing non-relapsing to relapsing patients, but demonstrated less NKp46 and DNAM-1 expressing NK cells (CD56^dim^ and CD56^bright^) in all patients after discontinuation, compared with healthy individuals [93]. Degranulation capacity towards the K562 cell line was preserved in both relapsing and non-relapsing patients compared with healthy donors, while IFN-γ and TNF-α production after stimulation were diminished in relapsing patients [95]. When dichotomized into high versus low IFN-γ and TNF-α production, patients with increased amounts of cytokine production were significantly associated with higher molecular relapse-free survival [95]. Mizoguchi et al. in agreement with this, have described a sustained elevation of NK effector IFN-γ^+^ cells in successful TFR after imatinib treatment (STOP-IM trial), suggesting that this cell population is essential for control of the leukemic clone in this setting [91].

Furthermore, homozygosity for KIR A haplotype conferred a significantly higher likelihood of achieving successful TFR in patients after imatinib or nilotinib treatment cessation as reported by Caocci et al. [96]. Conversely, patients with Bx genotype and KIR-ligand combination KIR2DS1/KIR3DL1 present/HLA-Bw4 present had a higher risk of relapse [96].

Altogether, these results demonstrate the importance of NK cells in control of the leukemic clone in the setting of TFR. Therefore, it may be reasonable to consider a therapy that enhances NK cell function as well as investigating NK cell specific markers before discontinuation of treatment.

### 4.2. Classical MPNs

#### 4.2.1. Immunological Changes in Classical MPNs

Typical untreated MPN presents with clonal myeloproliferation, decreased apoptosis of myeloid cells and in some cases progressive myelofibrosis [48]. MPN patients usually display an increased number of bone marrow nucleated-, terminally differentiated- and replicative mature cells and elevated peripheral blood cells. If left untreated, microvascular and major thrombotic events can occur [97]. The MPL mutation occurs in megakaryocytes, where it translates to a constantly activated MPL or thrombopoietin receptor [98], while the JAK2^V617F^ mutation occurs in multipotent hematopoietic progenitor cells, but can be found in all myeloid lineages, as well as B cells, T cells and NK cells [4]. It is unsurprising that several blood parameters are seen to be altered in the various MPN phenotypes. While it is rare to find a case of MPN with normal blood parameters, it is not impossible. The presence of the JAK2^V617F^ mutation with normal peripheral blood and T cell composition has previously been reported [97].

#### 4.2.2. NK Cells in Classical MPNs

**Figure 3:**
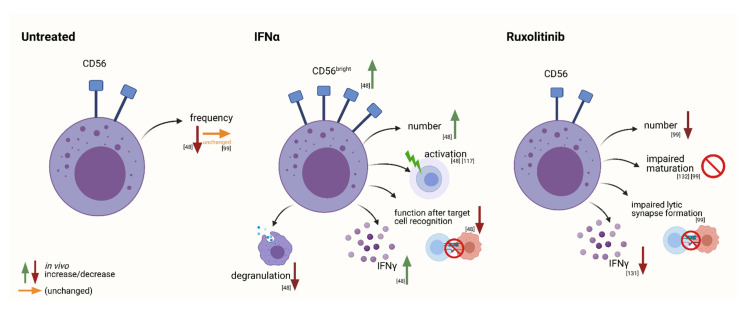
In vivo alterations to the NK cell compartment of Ph− MPNs before therapy initiation, as well as with IFNα or ruxolitinib therapy. Green arrows represent an increase in the frequency of specific properties observed, while red indicate a decrease or impairment, and yellow indicates no change. References are displayed in brackets. Created with BioRender.com (accessed on 15 June 2021).

Baseline data for NK cells in untreated MPNs remains disputed. Some studies report low NK cell frequencies in untreated patients (excluding therapeutic phlebotomy), compared with healthy donors [48], while we reported similar NK cells frequencies in untreated patients when compared with healthy age-matched controls (Figure 3) [99]. The phenotype of NK cells, however, appears to be most affected by pharmacological treatments such as IFN-α or TKI’s [48,99].

#### 4.2.3. The Effect of Treatment on NK Cells 

Therapeutic phlebotomy can be used in Ph− MPNs to maintain hematocrit levels, thereby reducing risk of thrombosis, and cardiovascular related death [100]. Studies regarding the effect of phlebotomy in MPN are lacking; however, patients undergoing regular phlebotomy for hereditary hemochromatosis, as well as healthy controls, showed no changes in absolute numbers of NK cells, B cells, T cells, NKT cells or monocytes [101]. Anti-coagulants can be used for antithrombotic therapy and typical forms of management of bleeding or infection may be indicated [18,19,21,102]. For the prevention of venous thromboembolism administration of vitamin K antagonists (VKAs) or the use of direct oral anticoagulants (DOACs) is recommended. Low-dose acetylsalicylic acid (ASA) may be indicated in several MPN entities with concomitant thrombocytosis, such as PV or ET [17,103,104,105]. Another treatment option is the administration of interferon-alpha (IFN-α) in a variety of MPN entities, such as PV and ET [9,10,18,19,105,106,107]. Cytoreduction is frequently accomplished via treatment with hydroxyurea (HU), an inhibitor of ribonucleotide reductase, or busulfan, an alkylating agent [19,21,108,109]. Anagrelide, a phosphodiesterase inhibitor, is approved for the reduction of platelet count in ET [19,109]. With *JAK2* mutations being the characteristic driver mutation in PV, ruxolitinib, a nonselective JAK1/2 inhibitor, is the option of choice in this entity, but can also constitute an alternative option in ET or PMF. [18,19,21,22,110] In situations where ruxolitinib has failed, or might not be appropriate, MF patients with anemia may be treated with immunomodulatory drugs (IMiDs) such as Thalidomide, Pomalidomide or Lenalidomide [111,112,113,114]. In high-risk PMF, CNL or CEL-NOS, certain patients may be even eligible for stem cell transplantation (SCT) [9,10,21]. 

##### Acetylsalicylic Acid (ASA)

Low dose ASA therapy is recommended to many MPN patients, particularly in ET, to reduce the risk of thrombotic events. ASA irreversibly acetylates platelet cyclooxygenase (COX)-1 resulting in a long-lasting inhibition of TXA2 biosynthesis, a type of thromboxane which stimulates platelet activation, contributing to vasoconstriction and platelet aggregation [115,116]. Recent data describing the effect of ASA on NK cells is lacking, and the effects in MPN specifically have not been reported.

##### Interferon-Alpha2

INF-α is a type 1 interferon which stimulates the immune cells, providing anti-proliferative, immunomodulatory, and antiangiogenic effects [117]. Pegylated IFN-α has a much longer serum half-life than recombinant IFN-α, and allows for weekly, as opposed to daily, dosing [118]. Type 1 interferons act by activating the JAK/STAT pathway, resulting in the production of a complex named IFN-Stimulated Gene Factor 3 (ISGF3), which translocates to the nucleus and initiates the transcription of hundreds of IFN-stimulated genes (ISGs) [119]. ISGs can perform an array of functions, including inhibition of viral spread, the downregulation of telomerase activity in malignant and non-malignant hematopoietic cells and the induction of a direct pro-apoptotic effect on myeloid progenitors [117,120].

Chronic IFN-α treatment depletes HSCs and has severe immune-altering effects including the activation of dendritic cells, NK cells and T cells [48,117]. MPN patients undergoing therapy with IFN-α displayed a significantly increased frequency of circulating Tregs. This is hypothesized to be due to either the mobilization of Tregs to the periphery, decreasing their immunosuppressive action within the bone marrow, or as a counter response to immune activation [121]. IFN-α treatment of MPN patients also resulted in a decreased frequency of myeloid DCs (mDCs) and plasmacytoid DCs (pDCs) [121]. This marked immunomodulation however, does not correlate with molecular responses, and the duration of treatment is also proposed to be a contributing variable [121].

Riley et al. 2014, described the effects of IFN-α treatment on NK cells in detail (Figure 3, IFN-α), in a comparison with untreated patients, healthy donors, and patients undergoing treatment with hydroxyurea [48]. During IFN-α treatment, NK cells increased in number, and underwent a phenotype shift from cytotoxic cells highly capable of antibody-dependent cellular cytotoxicity, to a more immune-stimulatory profile with little cytotoxic importance [48]. NK cell numbers were the lowest in untreated patients compared with healthy donors, or patients treated with hydroxyurea or IFN-α. An expansion of CD56^bright^ and subsequent decrease in CD56^dim^ NK cells was observed in long-term (≥12 months) IFN-α treated patients, and data suggests an overall decrease in NK cell functionality upon target cell recognition, and a shift from a more cytotoxic phenotype, as previously mentioned, to an immune-stimulatory profile. The CD56^dim^ subset, which is suggested to be a more mature subset, was seen to secrete an increased level of IFN-γ when stimulated with DC monokines (IL-12 and IL-15), however the overall production of IFN-γ was greater in CD56^bright^ cells, highlighting the dependence of NK cell differentiation on DC cytokines [48]. A decrease in degranulation and CCL4 production was seen during treatment, and while the observed trends were not significant, they support the conjectured compromised functionality [48] (Figure 3).

##### Hydroxyurea

Hydroxyurea is an antimetabolite which scavenges tyrosol-free radicals, thereby inhibiting the enzyme ribonucleotide reductase and reducing deoxyribonucleotide production, arresting proliferating cancer cells in the S-phase [106,108]. In a large retrospective study of 3411 patients immunomodulatory effects were not evident, as no allergy or immunosuppression was detected, and little has been reported in studies so far [106]. The bone marrow suppression and antiproliferative effect of HU may result in neutropenia, anemia or thrombocytopenia [122]. Upon activation, murine cells in culture that were treated with HU, increased IL-2 secretion, while no effect was seen in untreated cells [122], and similar results were seen in humans undergoing HU treatment for HIV infection [123]. HU was shown to upregulate NKG2D ligand expression on myeloid leukemic cell lines [124]; however, in MPN patients no changes were observed between HU treated patients and healthy controls [125].

##### JAK Inhibitors

For the treatment of Ph− MPNs, JAK inhibitors such as ruxolitinib, have been designed to specifically inhibit the JAK/STAT pathway by binding to cytoplasmic JAK1 and JAK2 kinases and modulating intracellular cytokine signaling [126]. The inhibition of JAK2 leads to the desired myelosuppression, while the inhibition of JAK1 reduces the levels of pro-inflammatory cytokines produced, improving various symptoms including bone marrow fibrosis [127,128]. A high rate of infection has been observed in patients undergoing ruxolitinib therapy, and it is hypothesized that the inhibition of JAK1 could therefore be responsible for a certain level of immunosuppression. Cytokine action on the JAK/STAT pathway results in proliferation, differentiation, and activation of various immune cells, however the full extent of ruxolitinib-induced immunosuppression is not yet known [129]. In both the COMFORT I and COMFORT II studies, neutropenia was seen in ruxolitinib treated patients compared with the placebo group (7.1 vs. 2% and 8.9 vs. 6.3% respectively) [129]. Several studies have shown that ruxolitinib treatment impaired dendritic cell function, affecting cell differentiation, tissue migration and IL-12 production, which has been described to play an important role in NK cell differentiation [107,129,130]. Later studies confirmed that IL-12 was completely blocked by ruxolitinib, as well as IL-15 and the phosphorylation of STAT5, resulting in a functional impairment of IFN-γ production by NK cells (Figure 3, ruxolitinib) [131]. An inhibition of cytokine secretion by macrophages was also reported, possibly effecting NK cell recruitment, maturation, and killing activity [132]. The reported impairment of DC function also resulted in the impaired induction of antigen specific T cell responses [130]. Pharmacological inhibitors of the JAK/STAT pathway have detrimental effects on NK maturation and as a result many patients experience infectious complications during treatment [99]. Unsurprisingly, ruxolitinib lead to a reduced number of NK cells, most likely due to impaired maturation as a result of the lack of DC cytokines, displayed by an increased ratio of immature/mature NK cells [99]. Additionally, compromised lytic synapse formation with target cells lead to a reduction in killing activity [99]. However, these results were reversible, and when ruxolitinib treatment was discontinued, NK cell function was restored [99] (Figure 3).

##### Immunomodulatory Drugs (IMiDs)

IMiDs are anti-inflammatory, anti-angiogenic drugs that regulate cytokine response. Even though IMiDs only play a minimal role in the management of MPNs clinically, they have substantial immunomodulatory activity on NK cell function [111,112,113,114]. MF patients treated with IMiDs exhibited activated NKT cells, co-stimulation of T cells, and impaired proliferation and function of Tregs [133]. More detailed effects of IMiDs on the immune cells can be seen in patients with multiple myeloma. In addition to an increase in IL-2 production and resultant T cell proliferation, a subsequent increase in IFNγ was seen, resulting in an increased number and improved function of NK cells [134]. The cytokines which are upregulated with thalidomide administration are also associated with angiogenesis, however the precise role of thalidomide and NK cells in vascularization has not yet been defined [135].

## 5. Discussion

The concepts of immune evasion and excessive inflammation are two frequently occurring tumor promoting phenomena [23,136]. Properly functioning immune surveillance is crucial for the detection and elimination of altered cell populations that could hypothetically transform into malignancies [136]. On the other hand, chronic hyperinflammatory conditions may additionally support tumor pathogenesis and exert tumor promoting effects [24,136]. Immunologic changes may therefore substantially affect the development and course of neoplastic diseases and constitute useful biomarkers for better understanding and prediction of pathologic processes.

Changes in immunologic variables were also discovered in the group of MPNs [25,26,33,137]. These alterations also comprise the NK cell compartment [27,48,54,55]. Changes in amount, phenotype as well as function of NK cells were reported to be present at time of diagnosis, but also occur with therapeutic intervention (Figure 1 and Figure 3) [55,99]. It appears that these findings are not mere observations but may also have prognostic value, especially in the setting of CML (Figure 2) [95].

In CML, conflicting results on relative amounts of NK cells as proportions among lymphocytes were demonstrated [34,65,66,67]. We have, however, previously shown that assumptions concerning NK cell frequencies should be regarded critically, and we proposed a gating strategy using whole blood for more accurate quantification by flow cytometry [65]. Using this method we demonstrated increased frequencies of NK cells at time of diagnosis [65].

Similarly, also in classical MPN entities, ambiguous findings concerning NK cell frequencies at time of diagnosis were reported, with reports of decreasing as well as increasing proportions [48,99]. However, evidence of changes in the NK cell compartment in untreated patients remains scarce, and a more in-depth investigation of the disease-related effects is needed. 

Additionally, phenotypic changes at time of diagnosis have been described in CML, mainly comprising a reduction of activating receptors [32,34,68]. These alterations may hypothetically lead to a more suppressed NK cell phenotype with a decrease in tumor cell recognition [32,63,70]. In agreement with this, a deficit in degranulation capacity of patient-derived NK cells at time of diagnosis was reported [66]. Additionally, the NK cells were shown to have a significantly reduced proliferative capacity, even though this finding could not be confirmed in the group’s mouse model [66].

The NK cell compartment may not only be altered by the disease itself, but the therapeutic agents used can also have immunomodulatory side effects (Figure 1 and Figure 3). Therapy of MPNs is heterogenous and the effects on NK cell functionality and phenotype vary substantially among the respective drugs. The effects of treatment on the NK cell compartment in Ph− MPNs is not easily summarized. One must consider the first line treatments for the various disease phenotypes, which, in many cases can be implemented throughout the course of disease until other factors such as age, mutational burden, or progression to fibrosis come into play. 

Therapeutic strategies in CML are majorly comprised of the group of TKIs. A variety of effects on NK cells have been reported, however, strong discrepancies especially between in vivo or ex vivo, and in vitro models make it necessary to carefully evaluate the respective findings. 

In vitro experiments on imatinib and nilotinib suggest negative effects on NK cell reactivity towards the leukemic clone [63,66,78,80]. Fortunately, however, the supposed decrease in functionality of NK cells conferred by imatinib as well as nilotinib application, was refuted in in vitro experiments using patient-derived material, suggesting that the suppressing effects of imatinib and the phenotypic alterations may not be of significant value for leukemic cell control [66,79,80]. When further investigating the in vivo effects of nilotinib therapy, on the other hand, Hayashi et al. found a decrease in cytotoxicity [79]. Even though this in vivo setting is probably depicting the situation more accurately, it would be of interest to investigate the demonstrated effects further by individually comparing patients pre-treatment to patients under treatment in order to reliably distinguish between disease-related and treatment-related effects. Another possible explanation for the ambiguous results is that nilotinib may not exert direct cytotoxicity-reducing effects, however it may indirectly modulate NK cells by changing the tumor environment. 

Dasatinib is probably the most substantially studied TKI in terms of NK cell related effects in CML therapy. One of the observed drug-specific effects is the well-studied expansion of LGLs in vivo with simultaneous augmentation of NK cell counts [32,81,82,83]. This appears to be a direct effect of dasatinib application on NK cells, as in vitro use of dasatinib on healthy cells reportedly led to an increase in NK cell counts [84]. Concerning functional effects of dasatinib administration, it is important to clearly distinguish between direct and indirect effects. Direct administration of dasatinib led to a reduction of cytotoxicity, degranulation and cytokine secretion of NK cells in vitro [80,85]. However, when dasatinib was washed out after the application, the effects were contrary with an increase in proliferation as well as cytolytic capacity [85,86]. Most importantly, in vivo experiments examining patients under dasatinib treatment confirmed the increase in cytotoxic capacity of NK cells [79]. The stimulatory effects of dasatinib may therefore best reflect the actual effects. However so far, the in vitro results do not provide enough information to clearly distinguish between a direct effect of dasatinib application or indirect stimulation of NK cells.

Fully functioning NK cells are important players in leukemic immune surveillance in CML. This is also reflected by the prognostic value they exert for molecular response. An increase in NK effector cells, as well as weak linkage of inhibitory KIR/HLA combinations, were associated with superior outcome, as well as increasing NK cell counts [83,89,90,91,96]. These findings indicate that changes in the NK cell compartment under TKI therapy may actively influence sensitivity towards the treatment, making investigations into drug-specific alterations even more important.

The role of NK cells in CML becomes even more evident in investigations on patients after TKI treatment when the individual’s immune system takes control over of the leukemic clone. The amount of NK cells at the time of treatment cessation is an important prognostic factor in the setting of imatinib and dasatinib cessation [94,95]. Furthermore, preserved NK cell production of IFN-γ and TNF-α was associated with successful TFR after imatinib discontinuation, as well as an increase in the IFN-γ producing NK cell effector population [91,95].

Treatment with IFN-α is common in classical MPNs, and while it has vanished from the therapeutic landscape of CML treatment with the introduction of TKIs it is now being discussed as a possible add-on therapy. An expansion of the CD56^bright^ population was seen in both classical MPNs as well as Ph+ MPNs with IFN-α [48,76]. This shift was suggested to constitute a maturation defect [48]. In CML patients, however, favorable effects of IFN-α, such as an increase in NK cell proportions, as well as increased numbers of mature and activated NK cells, and an upregulation of cytotoxicity were described [73,74,75,76].

The effect of HU on NK cells has not been thoroughly investigated and lacks in-depth analyses. However, currently available data suggest that there are no detectable changes in the NK cell compartment with HU treatment [125,138,139].

Ruxolitinib had drastic effects on the overall immune system, the JAK1 inhibition specifically, caused by ruxolitinib, is hypothesized to be responsible for a certain level of immunosuppression [129]. Ruxolitinib affected the NK cell compartment both directly and indirectly. Direct effects include a reduction in number of NK cells and a greater proportion of immature NK cells, as well as a reduction in killing activity due to the reduced ability to form lytic synapses with target cells [99]. Indirect effects include the impairment or complete blocking of dendritic cell IL-12 and IL-15 production, or the phosphorylation of STAT5, resulting in defective maturation and a diminished functional capacity of NK cells to produce IFN-γ [107,129,130,131]. The reduction in NK cell number is hypothesized to be a result of the impaired maturation. Indeed, we reported a 2.5 fold higher bright:dim ratio in untreated patients than in aged-matched healthy donors, indicative for a block in differentiation [99]. Many aspects of lymphoid cell development and homeostasis are controlled by cytokines, and as MPNs are diseases driven by inflammation [27], it is no surprise that this hyperinflammatory state can contribute to maturation defects and dysregulation of homeostasis [140]. Interestingly, studies investigating the effects of ruxolitinib treatment in patients with STAT1 gain-of-function mutations reported higher levels of STAT5 phosphorylation post-treatment, and the perforin expression appeared to be restored, contradictory to the results reported in MPN patients [141]. Vargas-Hernandez and colleagues suggested this could be a result of the degree of phosphorylation, with STAT1 levels elevated during IL-2 stimulation, while STAT5 was aberrantly phosphorylated, as both STAT1 and STAT5 were affected to the same degree of impairment [141]. The in-depth and specific effects of ruxolitinib on the NK cell phenoptype in Ph− MPNs, however, are hard to definitively conclude, as supporting literature and studies are limited.

Overall, the impact of NK cells in MPNs is well established and substantiated in CML. NK cells play a role in control of the leukemic clone which is not only reflected by their prognostic impact in the setting of TFR, but also important for the achievement of molecular remission under TKI therapy (Figure 2). The number of NK cells present, sufficient cytokine production, as well as expression of activating NK cell receptors with matching HLA-counterparts appear to be important prognostic features for molecular remission in CML. Interestingly, in contrast to CML, research on NK cells in classical MPNs still substantially lacks information at all stages of disease. The major questions to be addressed, concern alterations in the NK cell compartment at time of diagnosis, as well as most importantly, their prognostic impact. With the known suppressive effects of ruxolitinib administration on NK cell function, including impaired lytic synapse formation, and reduced recruitment, activation and killing activity (Figure 3), it would be of primary importance to thoroughly investigate this finding in terms of patient outcomes and whether patients may profit from additional NK-cell specific therapy.

NK cell activity may be influenced with the use of checkpoint-inhibitors, most of which so far have only been investigated in vitro [142]. One of the targets which could potentially influence NK cell reactivity and has been implemented in a variety of cancer entities, is PD-1 with its counterpart PDL-1 [142]. PD-1 blockade is a well-established mechanism of immune evasion and has already been discussed as a possible therapeutic target in CML [143,144]. Further research on these inhibitors in vitro and in vivo would thus certainly be of interest, especially in the field of CML, and could potentially ameliorate sensitivity towards TKI treatment or successful TFR through upregulation of NK cell activity. 

## 6. Conclusions

Regardless of the amount of research currently documented on NK cells in MPNs, the story does not end here. In particular, the lack of data concerning classical MPNs and the NK cell population support the need for more in-depth studies. Furthermore, we believe that evidence of the great prognostic impact of NK cells in CML suggests that it is time to target NK function, hopefully improving control of the leukemic clone for a deeper molecular response and more sustainable treatment free remission.

## Data Availability

Data sharing not applicable. No new data were created or analyzed in this article.
